# A Systematic Review About Postmortem Pink Teeth: Forensic Classification, Diagnostic Value, and Analysis Methods

**DOI:** 10.3390/diagnostics15162092

**Published:** 2025-08-20

**Authors:** Isabella Aquila, Saverio Gualtieri, Aurora Princi, Matteo Antonio Sacco

**Affiliations:** Institute of Legal Medicine, Department of Medical and Surgical Sciences, University “Magna Graecia” of Catanzaro, 88100 Catanzaro, Italy; saveriogualtieri@icloud.com (S.G.); aurora.princi@studenti.unicz.it (A.P.); matteosacco@unicz.it (M.A.S.)

**Keywords:** postmortem interval, hemoglobin diffusion, forensic odontology, postmortem changes, dental discoloration

## Abstract

**Background:** The phenomenon of pink teeth represents a notable observation in forensic science, although its interpretation remains complex and not directly attributable to a specific cause of death. **Methods:** This systematic review provides an updated and comprehensive overview of the morphological and histological mechanisms associated with this finding, with a focus on hemoglobin diffusion and pigment accumulation during putrefaction rather than on detailed biochemical pathways. **Results:** Environmental conditions, especially high humidity and moderate temperatures, are identified as key facilitators. The synthesis of the available evidence, including case reports, observational series, and experimental studies, confirms that pink discoloration is primarily linked to postmortem hemoglobin diffusion following erythrocyte breakdown and release of heme groups into dentinal structures. This process occurs more frequently under conditions that preserve hemoglobin and facilitate its migration into dental tissues. Importantly, pink teeth have been documented across a wide spectrum of postmortem scenarios, such as hanging, drowning, carbon monoxide poisoning, and prolonged exposure to humid environments, indicating that their presence is neither pathognomonic nor exclusively associated with a specific cause of death. Assessment methods include semi-quantitative visual scoring systems (e.g., SPTC and SPTR), spectrophotometric assays, and histochemical analyses for hemoglobin derivatives. Recent advances in digital forensics, particularly micro-computed tomography and artificial intelligence–based segmentation, may further support the objective evaluation of chromatic dental changes. **Conclusions:** This review underscores the need for standardized approaches to the identification, classification, and analysis, both qualitative and quantitative, of pink teeth in medico-legal practice. Although not diagnostic in isolation, their systematic study enhances our understanding of decomposition processes and contributes supplementary interpretive data in forensic investigations.

## 1. Introduction

The postmortem phenomenon known as “pink teeth” refers to a pinkish discoloration of dental tissues that can occur during the decomposition process. This phenomenon is characterized by a reddish or pink hue most commonly observed in the cervical dentin region and has long attracted attention in forensic science due to its striking appearance and its capacity to generate interpretative challenges in medico-legal investigations. The earliest documented cases trace back to the 19th century, when early forensic practitioners first noted the unusual coloration during routine autopsies. Since that time, the modern forensic literature has increasingly focused on its morphology, histological features, and potential evidentiary relevance, underlining the importance of properly documenting and interpreting such findings within a rigorous scientific framework [1,2].

The coloration is generally believed to result from the infiltration of hemoglobin and its breakdown products into dentinal tubules. This process occurs most typically in early putrefaction, when hemolysis and increased tissue permeability allow pigments to diffuse progressively into hard dental structures, leading to their characteristic appearance [3,4]. Pink discoloration is most often observed in anterior teeth, particularly the incisors and canines, and has been associated with various death contexts, such as asphyxia, drowning, freezing, and carbon monoxide poisoning [5,6,7]. However, it is important to acknowledge that the literature also reports occurrences in cases unrelated to asphyxial deaths, reinforcing the notion that the phenomenon is influenced by a multitude of factors rather than a singular etiology.

Despite numerous reported observations, the forensic significance of pink teeth remains controversial and is often debated among forensic odontologists and pathologists. Several authors have emphasized that the phenomenon is not directly linked to any specific cause of death, nor is it consistently present across all cases of similar circumstances or environmental exposure [8,9]. Factors such as environmental humidity, body positioning, ambient temperature, and individual dental anatomy all appear to interact in complex ways that influence the development, intensity, and distribution of the discoloration [10]. This multifactorial interplay underscores the necessity of integrating pink teeth findings within a broader context of autopsy results and scene investigation.

Forensic studies suggest that pink teeth may serve as a supplementary indicator of early decomposition or of specific environmental conditions to which the body has been exposed prior to discovery. However, the interpretation must always remain cautious and conservative, as various confounding factors—such as bacterial contamination, medication-induced pigmentation, and cervical resorption in living individuals—may produce similar appearances that risk being misclassified if assessed in isolation [11,12]. Consequently, comprehensive histological and spectrophotometric analyses, when available, are essential to increase diagnostic accuracy.

This review aims to summarize the current understanding of the pink teeth phenomenon by examining its structural basis, associated death conditions, and environmental influences. In particular, special attention is given to the limitations of existing classification and analytical methods and to the need for standardized protocols to enhance its forensic application and reproducibility. While not pathognomonic, pink teeth remain a noteworthy and visually evident postmortem feature that, when contextualized within autopsy findings and interpreted with due caution, can contribute useful supplementary information to medico-legal investigations, especially in cases involving prolonged environmental exposure.

## 2. Materials and Methods

This review was conducted to synthesize and integrate current knowledge about the phenomenon of postmortem pink teeth, with particular attention to its forensic significance, histopathological underpinnings, and potential diagnostic implications in routine practice. This review was designed to answer two core forensic questions: (1) What is the pathophysiological basis and diagnostic relevance of pink teeth? and (2) What methods can be used to detect, analyze, and interpret this finding in a reproducible and standardized manner? To address these questions, we conducted a structured review of the literature using predefined criteria and quality appraisal tools. A structured literature search was performed in PubMed, Scopus, Web of Science, and Google Scholar using the Boolean expression (“pink teeth” OR “pink tooth” OR “postmortem tooth discoloration”) AND (“forensic” OR “autopsy” OR “decomposition”). The search included all records published up to 30 June 2025. A total of 524 records were retrieved across all databases, comprising 448 results from Google Scholar, 41 from Scopus, and 35 from PubMed. The search on Web of Science yielded a limited number of overlapping entries and was used primarily to cross-reference citations. Due to the inclusive nature of Google Scholar, its results required manual filtering to exclude non-peer-reviewed publications, conference abstracts, and grey literature. Only articles written in English were considered eligible for full-text evaluation to ensure accessibility and uniformity of content. All records retrieved from the databases were imported into a reference management tool, and duplicates were eliminated. Two independent reviewers screened the remaining studies at the title and abstract level. The full texts of potentially eligible articles were then assessed according to predefined inclusion and exclusion criteria. Studies were considered eligible if they described postmortem cases with documented pink tooth discoloration, provided data on the underlying histological or physico-chemical mechanisms, or proposed methods for detection, classification, or analysis applicable to forensic pathology. Case reports, retrospective case series, reviews, experimental animal studies, and studies involving radiological or microscopic simulations were included when pertinent to the research aims and objectives [1,2,3,4,5,6,7,8,9,10,11,12,13,14,15,16,17,18,19,20,21,22,23,24,25,26,27,28,29]. Excluded were articles not published in English, works without a peer-review status, inaccessible full texts, and studies unrelated to forensic relevance, such as those dealing with cosmetic or dental restoration aspects.

**Figure 1:**
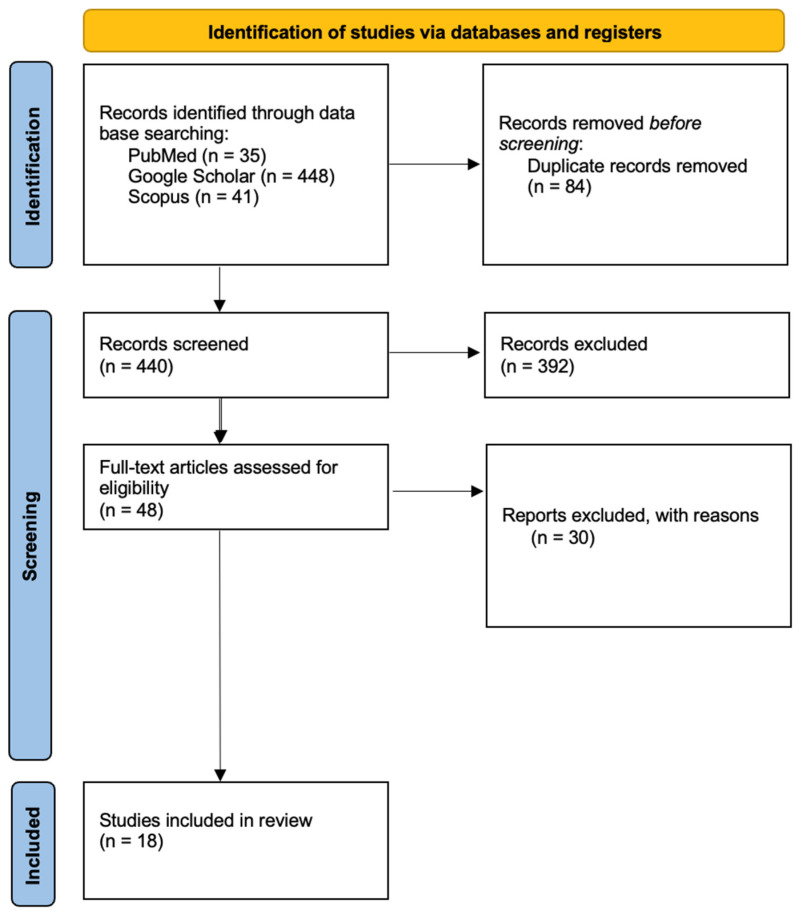
PRISMA flowchart.

After the removal of 84 duplicates, 440 unique records were screened by title and abstract. Of these, 392 were excluded for lack of relevance, methodological insufficiency, or non-peer-reviewed format. Forty-eight full-text articles were assessed for eligibility, and thirty were subsequently excluded due to insufficient data, irrelevance to forensic application, or inaccessibility. Eighteen studies were ultimately included in the qualitative synthesis: thirteen with direct forensic focus (Table 1) and five with ancillary or contextual relevance (Table 2) (Figure 1).

To evaluate the methodological robustness of the included studies, a critical appraisal was carried out using the Joanna Briggs Institute (JBI) Checklists appropriate for each study design. Each study was assessed independently by two authors, and any discrepancies were resolved by consensus. The JBI criteria were applied to evaluate aspects such as clarity of objectives, adequacy of case description, validity of measurements, and reliability of results. The studies were then stratified into three levels of quality: high, moderate, and low, corresponding to total scores of ≥80%, between 60 and 79%, and <60%, respectively. Each study was scored on clarity of methods, forensic relevance, internal validity, reproducibility, and robustness. Studies scoring 8–10 points were classified as high quality (*n* = 9), 6–7 as moderate (*n* = 6), and ≤5 as low (*n* = 3).

The main variables extracted from each study included the year of publication, type of study design, cause of death (if applicable), environmental or postmortem conditions reported, and primary findings relevant to pink discoloration. The articles were then systematically classified into two tables: one for the primary forensic literature directly addressing the postmortem pink teeth phenomenon and another for contextual or ancillary studies offering indirect or supportive insight into its interpretation.

Discrepancies in classification or interpretation between the reviewers were resolved by consensus discussion in line with established protocols for systematic review methodology. Where necessary, articles cited by included studies were also retrieved and reviewed in order to ensure comprehensive coverage and minimize the risk of omitting relevant contributions. This approach facilitated a more nuanced synthesis of the available evidence base.

To ensure a focused and coherent synthesis of findings, we explicitly excluded studies that did not contain direct or contextual references to the phenomenon of pink teeth. Specifically, we did not include articles centered solely on aesthetic dental procedures, restorative materials, or oral pigmentation of non-forensic origin, as these fall outside the forensic scope of the review.

## 3. Results

An extensive review of the literature yielded a total of 18 studies addressing the phenomenon of postmortem pink teeth and their implications for forensic practice. These studies were classified into two categories based on the nature, quality, and forensic relevance of the investigation: (i) direct forensic studies focused specifically on postmortem pink teeth, presented in Table 1, and (ii) ancillary or contextual studies where pink teeth were either secondary findings or analogically discussed in clinical or radiological contexts, presented in Table 2. This division allowed for a more structured appraisal of the evidence.

In Table 1, the 13 selected forensic studies include case reports, observational studies, experimental studies, and reviews. Among them, Gowda et al. [1] and Soriano et al. [7] provided isolated case reports involving pink teeth identified in forensic autopsies following asphyxial deaths, highlighting the diversity of contexts in which the phenomenon may occur. Stavrianos et al. [11] and Franco et al. [12] supported the role of hemoglobin diffusion from pulp tissue into dentinal tubules as a potential explanatory mechanism, thereby reinforcing the hypothesis of endogenous pigment infiltration. Experimental works by Van Wyk [13] and Sainio et al. [21] further highlighted the role of blood dynamics, gravitational hypostasis, and the timing of hemolysis in color formation. Campobasso et al. contributed a large observational dataset derived from cadavers recovered from prolonged aquatic immersion, observing age-related susceptibility and pigment deposition patterns consistent with hemoglobin derivatives [22]. Brøndum and Simonsen identified pink discoloration in 26 of 119 forensic cases, suggesting an incidence of 21.8% without significant correlation to any specific cause of death [23]. Notably, Brites et al. suggested the importance of environmental factors, including sustained humidity, in facilitating the appearance and distribution of pink coloration [24]. Minegishi et al. presented the largest observational series to date, documenting 68 cases of pink teeth among 324 unidentified cadavers [25]. Their findings confirmed a higher prevalence in anterior teeth and in individuals under the age of 60, while no significant association was observed with sex, cause of death, or location of discovery, reinforcing the hypothesis that the phenomenon is nonspecific and environmentally mediated [25]. Sakina Tri Meilana et al. reported two well-documented cases involving decomposed bodies exhibiting pink discoloration, emphasizing the importance of environmental moisture and decomposition in shaping the appearance of the teeth [15]. Similarly, Pasaribu et al. described the pink teeth phenomenon in two victims found in humid but non-submerged indoor environments, suggesting that high ambient humidity alone may be sufficient to trigger the discoloration process [16]. Braga et al., in a narrative review, confirmed that pink teeth are most frequently observed in cases of immersion, strangulation, or carbon monoxide poisoning, but concluded that no direct causative link exists [5]. The authors also highlighted the selective involvement of anterior single-rooted teeth and younger individuals, echoing patterns observed in previous reports.

In Table 2, five ancillary studies were included. These did not originate from forensic casework per se but were nonetheless considered to contribute interpretative relevance by drawing attention to possible differential diagnoses. Heithersay [4,6], in particular, discussed invasive cervical resorption and related dentin-pulp changes that can lead to discoloration resembling postmortem findings, underscoring the potential for misinterpretation. Ataseven et al. conducted an experimental study on rats drowned in water, revealing statistically significant postmortem histological changes in the dental pulp—such as edema, vascular congestion, and odontoblast detachment—compared to controls [18]. These findings suggest that pulp analysis may contribute supportive evidence in drowning investigations. A historical review by Sakurada revisited the extensive experimental work of Kato (1941), in which 203 rabbits and 4 human cases were examined following various types of cervical compression [19]. Notably, pink teeth were observed even in early postmortem stages before decomposition, suggesting a possible link with vascular or mechanical factors rather than putrefaction alone. Finally, Dye et al. reported pink discoloration in archaeological specimens from medieval skeletons [20]. Despite superficial similarity to the forensic phenomenon, the analytical results indicated a different etiology—likely fungal in origin—underscoring the need for differential diagnosis when interpreting pink teeth in long-term postmortem or archaeological contexts. So, in the context of forensic investigations, the interpretation of postmortem discoloration can be complex, and it is essential to explore multiple potential causes.

Across the reviewed literature, the frequency of pink teeth ranged from 5% to 29% of the examined cases, with anterior teeth more commonly affected than molars. In the Minegishi series, 68/324 unidentified cadavers (21%) exhibited pink discoloration, most prominently in individuals under 60 [14]. No significant correlation emerged between pink teeth and sex, cause of death, or location of discovery, supporting the hypothesis of multifactorial, nonspecific causation. Confounding variables included environmental humidity, decomposition time, and antemortem dental pathology. Several ancillary conditions known to produce pink dental discoloration in vivo were also reviewed. These include invasive cervical resorption, internal hemorrhage, and chronic pulpitis. While not directly relevant to postmortem phenomena, such entities represent potential diagnostic confounders during forensic assessments. Overall, the phenomenon appears multifactorial, with contributions arising from hemolysis, environmental moisture, anatomic predisposition, and postmortem lividity dynamics. While frequently reported in forensic contexts involving decomposition or aquatic recovery, pink teeth do not appear to be pathognomonic of any specific cause of death. Environmental humidity and prolonged submersion in water are consistently reported to promote the diffusion and retention of hemoglobin and its derivatives within the dentinal tubules, facilitating the pink appearance that is often visually striking. Pigmented bacterial biofilms—especially those produced by chromogenic microorganisms—have also been suggested as potential confounding elements, though histological and spectrophotometric analyses typically support a hemoglobin-based origin rather than microbial pigmentation.

## 4. Discussion

### 4.1. Forensic Significance of Pink Teeth

The pink discoloration of teeth represents a noteworthy but nonspecific postmortem phenomenon that has repeatedly attracted the attention of forensic specialists due to its distinctive macroscopic appearance. It results from the infiltration of hemoglobin and its degradation products into the dentinal tubules, particularly in the cervical region, which appears to be more permeable and prone to pigment accumulation compared to other dental zones [1]. This finding, while visually striking and frequently remarked upon in autopsy reports, must be carefully contextualized within the broader autoptic framework that considers all aspects of decomposition and the postmortem interval. While visually distinctive, pink teeth do not permit definitive conclusions about the cause of death, as confirmed by studies showing their presence across heterogeneous fatal mechanisms [2,14,15,16].

Pink teeth have been observed in a wide variety of circumstances—including asphyxia, drowning, carbon monoxide poisoning, gunshot wounds, hypothermia, and natural deaths—thus demonstrating the lack of specificity that characterizes the phenomenon [3,11]. However, this very diversity in reported contexts confirms the absence of diagnostic exclusivity and underscores the need for a cautious, integrative approach to interpretation. Instead of serving as a primary indicator, the presence of pink teeth may offer indirect or circumstantial information about the postmortem environment and the stage of decomposition, especially when considered alongside other findings such as tissue maceration, hypostasis, and insect activity [4,16].

### 4.2. Morphological and Environmental Influences

A range of studies has explored in detail the numerous factors influencing the appearance and extent of pink teeth in cadaveric specimens. The principal chromophore responsible for the discoloration is hemoglobin, which infiltrates the dentin following erythrocyte breakdown. Earlier theories attributing a significant role to red pigment-producing bacteria have been largely refuted through controlled experimental and histological investigations that consistently demonstrated the presence of hemoglobin degradation products [3].

Environmental factors—including high humidity, prolonged submersion in water, and moderate ambient temperatures—are known to favor hemoglobin preservation and diffusion into dental structures. For example, Borrman et al. observed that pink teeth occurred more frequently in bodies recovered from aquatic settings, likely due to the stabilizing effect of water immersion on hemoglobin and the facilitation of its movement into dentinal tubules [2]. These observations indicate that while environmental conditions can enhance the phenomenon, they are not an indispensable prerequisite.

### 4.3. Incidence and Interpretative Evolution

Over the past decades, the phenomenon of postmortem pink teeth has been increasingly reported in the forensic literature, yet documentation remains fragmented and heterogeneous. Our updated search strategy retrieved more than 500 potentially relevant records across multiple databases (448 in Google Scholar, 41 in Scopus, and 35 in PubMed), highlighting sustained scholarly interest but a lack of comprehensive syntheses or standardized incidence reporting. The most extensive case series to date is that of Minegishi et al., who documented 68 cases of pink teeth among 324 unidentified autopsied cadavers—yielding a prevalence of approximately 21% [25]. Notably, pink discoloration was more frequently observed in individuals under the age of 60 and did not correlate significantly with sex, cause of death, or location of body discovery.

Such variability reinforces the view that pink teeth are neither rare nor diagnostically exclusive. Earlier studies, such as Van Wyk, focused on the in vitro timing of macroscopic color development linked to hemolysis during decomposition [13]. More recent contributions have expanded the focus to include histopathological and environmental variables. For instance, Pasaribu et al. [16] and Ataseven et al. [18] describe pink teeth in bodies found in humid environments or following drowning, suggesting that tissue hydration and postmortem interval may facilitate hemoglobin diffusion into dentinal tubules. Furthermore, Sakurada [19] reexamined the historic experimental work of Kato (1941) [19], proposing that pink discoloration may occasionally occur even in early postmortem stages, particularly following cervical compression and asphyxial deaths, thereby challenging the assumption that decomposition is always a prerequisite.

### 4.4. Methodological Approaches and Analytical Protocols

Histological, chemical, and spectrophotometric methods are essential in validating pink discoloration. Thin-layer chromatography and UV spectrophotometry have proven effective in detecting hemoglobin-derived pigments in dentinal tissue [7]. Franco et al. [12] highlighted the importance of proper sampling and evidence documentation, recommending the use of double gloves and DNA contamination precautions during autopsy.

Various authors have also proposed scoring systems for pink teeth analysis, such as the Score for the Degree of Discoloration (SPTC) and the Score for the Pattern or Region (SPTR). These tools aim to reduce subjectivity in visual evaluation, but their use is not yet widespread or standardized.

Advanced decomposition, dental restorations, medications, and even resorptive dental pathology may confound visual interpretation. These factors require differentiation through histological analysis, which is not always available in routine forensic contexts [17,18,19,20,21,22,23,24,25,26,27,28,29,30,31,32,33,34,35,36,37]. The lack of reproducibility and cost-intensive protocols further limit their applicability [23]. Recent technological advancements in forensic odontology underscore the potential of digital imaging and artificial intelligence to refine postmortem dental analysis. In parallel, technological advances have begun to reshape the interpretative landscape. Thurzo et al. demonstrated the utility of micro-computed tomography (micro-CT), cone-beam CT, and artificial intelligence–based segmentation for the reconstruction of dental patterns after acid exposure [38,39]. Although their study did not specifically address pink teeth, their interdisciplinary methodology suggests a promising framework for digitally quantifying postmortem pigment diffusion and for standardizing chromatic assessment across cases. These approaches may ultimately enhance diagnostic reproducibility and mitigate the subjectivity that currently limits the forensic utility of pink teeth.

### 4.5. Classification Challenges and Confounding Conditions

The classification of pink teeth remains a significant challenge. There is no universally accepted classification system that accounts for color intensity and anatomic distribution.

In addition to postmortem factors, several antemortem processes may generate similar discoloration. Heithersay et al. [4,6] demonstrated that invasive cervical resorption and internal pulp hemorrhage during life can result in pink coloration. Stavrianos et al. [11] further reinforced the concept that hemolysis is a prerequisite for pink tooth development, supporting Van Wyk’s in vitro study [13] that demonstrated the appearance of discoloration from day 6 postmortem onward.

Bacterial colonization—especially from anaerobic strains producing porphyrins—has also been implicated in false-positive interpretations, underscoring the need for interdisciplinary collaboration among forensic odontologists, microbiologists, and pathologists.

### 4.6. Interpretative Limitations and Practical Use

Despite their suggestive appearance, pink teeth should not be considered a reliable marker of postmortem interval or of specific causes of death. Soriano et al. [7] emphasized the wide variability of its appearance, which depends heavily on environmental factors rather than biological ones.

Thus, although some authors propose further standardization of analysis and classification [29], the practical utility of pink teeth in forensic identification remains limited. This reinforces the necessity of a multidisciplinary approach in interpretation, integrating morphological, histological, microbiological, and environmental data.

### 4.7. Pink Discoloration in Living Subjects

Pink discoloration of teeth is not exclusively a postmortem occurrence. Several antemortem conditions can cause similar chromatic alterations, potentially leading to diagnostic confusion during forensic examinations. Among these, intrapulpal hemorrhages following trauma or endodontic procedures, internal or invasive cervical resorption, and certain systemic diseases such as hemolytic anemia or chronic liver failure are most frequently implicated [4,23,24].

In particular, Heithersay et al. [4,6] demonstrated that invasive cervical resorption may result in a reddish discoloration that can be misinterpreted as a postmortem finding, especially in exhumed or decomposed remains. The authors highlighted how traumatic injuries to the pulp can lead to hemoglobin release and pink coloration, mimicking early postmortem phenomena. These insights underscore the importance of thorough dental and medical history analysis, whenever available, and of histological confirmation to differentiate between vital and postmortem origins of discoloration.

### 4.8. Overlap Between Antemortem and Postmortem Discoloration

Cases where death occurs shortly after dental trauma, internal hemorrhage, or pathological resorptive processes may present a diagnostic overlap between vital and postmortem pink discoloration [25,26,27,28,29,30,31,32,33,34,35,36,37,38,39]. In such instances, both mechanisms—active intravital bleeding and passive postmortem hemoglobin diffusion—may contribute to the observed chromatic pattern.

Van Wyk [13] demonstrated in a controlled in vitro study that pink discoloration becomes macroscopically evident approximately six days postmortem, only after the occurrence of hemolysis. Similarly, Stavrianos et al. [11] confirmed that true pink tooth development requires prior hemolysis and erythrocyte breakdown. Therefore, histological differentiation, especially with special staining techniques to detect intact versus lysed erythrocytes, remains the gold standard for distinguishing between these two origins.

In forensic practice, it is essential to critically evaluate pink discoloration in the broader context of dental anatomy, decomposition stage, environmental exposure, and known medical or dental history. Without such a comprehensive assessment, the risk of misinterpretation remains significant, potentially leading to erroneous conclusions regarding the timing or cause of death.

## 5. Conclusions

The pink teeth phenomenon, though visually striking and historically noted in forensic practice, must be interpreted with caution. The current literature consistently affirms that pink discoloration is not specific to any particular cause of death, nor is it a reliable marker for estimating the postmortem interval. Although several case series exist, no meta-analytical synthesis currently permits robust inference regarding causality or specificity. Despite the frequent suggestion of environmental causation, the current evidence remains observational and largely qualitative. Its occurrence is primarily influenced by environmental factors, particularly moisture and moderate temperature, which facilitate the diffusion of hemoglobin degradation products into the dentinal tubules during decomposition.

Despite its lack of diagnostic specificity, the presence of pink teeth may offer supplementary insights into postmortem environmental exposure, especially in humid or aquatic contexts. However, similar discolorations may also result from intravital conditions, such as internal resorption, pulp necrosis, or trauma, underscoring the necessity of differential diagnosis.

Current methodologies for documenting and analyzing pink teeth suffer from variability and limited standardization. Visual scoring systems remain subjective, while biochemical and histological techniques, though more reliable, are not routinely implemented due to resource constraints. A multidisciplinary approach that integrates forensic odontology, histopathology, and microbiological analysis is essential to improve the accuracy of interpretations. Emerging technologies, such as micro-CT, chemometric tools, and AI-based segmentation, may provide a future path toward quantification and reproducibility. However, until such approaches are validated and broadly implemented, pink teeth should be considered a suggestive but ancillary finding, whose interpretative value resides within a multimodal, context-aware forensic approach.

Further research is warranted to refine protocols, validate classification systems, and clarify the histochemical progression of the phenomenon. While pink teeth should not be considered a definitive forensic sign, a structured and standardized analysis may enhance their value as supportive findings in forensic investigations, provided that contextual variables and potential confounders are carefully considered.

## Figures and Tables

**Table 1:** Forensic studies directly focused on the postmortem pink teeth phenomenon.

Authors	Year	Study Type	Cause of Death	Environmental Conditions	Main Findings	Ref.
Gowda et al.	2015	Case Report	Asphyxia	High Humidity	Pink discoloration unrelated to specific cause of death	[1]
Soriano et al.	2009	Observational Study	Drowning, Strangulation	Wet Environment	Observed in asphyxia but not pathognomonic	[7]
Van Wyk	1988	Experimental Study	Not Applicable	Controlled In Vitro	Pink color appears around day 6 postmortem due to hemolysis	[13]
Stavrianos et al.	2011	Review	Multiple	Various	Postmortem hemoglobin diffusion theory supported	[11]
Brites et al.	2020	Observational Study	Mechanical Asphyxia	Humid	PT not specific to asphyxia; environmental link emphasized	[24]
Brøndum & Simonsen	1987	Case Report	Various	Not Stated	Pink discoloration described without environmental context	[23]
Franco et al.	2019	Review	Various	Various	Dental findings relevant to forensic context	[27]
Campobasso et al.	2006	Observational Study	Various	Prolonged seawater immersion	Pink teeth in 18 cases; more evident in younger individuals; pigment consistent with hemoglobin and derivatives	[22]
Sainio et al.	1990	Experimental + Literature Review	-	Controlled postmortem storage	Pink teeth due to hemoglobin derivatives; gravity-influenced distribution; more physical than chemical mechanisms	[21]
Braga et al.	2024	Narrative Review	CO, immersion, strangulation	Moist environments	No causal link; more frequent in anterior teeth and younger subjects	[5]
Sakina Tri Meilana et al.	2025	Case Report	Drowning, trauma, decomposition	Water immersion, decomposition	Two PTP cases; emphasized PMI and moisture; multidisciplinary interpretation advised	[15]
Pasaribu et al.	2024	Case Report	Not specified (non-drowning)	Indoor, highly humid, decomposed	PTP in non-submerged humid settings; supports environmental role; no diagnostic value for cause of death	[16]
Minegishi et al.	2022	Observational Study	Various	Mixed (indoors, outdoors, water)	68/324 cadavers showed PTP; anterior teeth were most affected; no correlation with cause of death or place of discovery	[14]

**Table 2:** Ancillary or contextual studies related to pink teeth (non-primary forensic focus).

Authors	Year	Study Type	Cause of Death	Environmental Conditions	Main Findings	Ref.
Heithersay	1999	Clinical Study	Not Applicable	Not Applicable	Cervical resorption can cause pink discoloration	[6]
Heithersay	2007	Clinical Study	Not Applicable	Not Applicable	Invasive resorptions mimic PM pink teeth	[4]
Dye et al.	1995	Archaeological Case Study	Not Applicable	Long-term burial (medieval)	Pink discoloration is likely fungal in origin, not hemoglobin-related	[20]
Ataseven et al.	2025	Experimental (rat model)	Drowning (animal model)	Aquatic + time-controlled	Histopathological pulp changes observed postmortem; potential marker in experimental drowning cases	[18]
Sakurada	2023	Historical Review	Asphyxia (animal + human)	Minimal decomposition	Early-stage PTP observed after cervical compression; suggests vascular mechanism independent of decay	[19]

## Data Availability

Not applicable to this article, as no datasets were generated.
